# Neutrophil properties in healthy and *Leishmania infantum*-naturally infected dogs

**DOI:** 10.1038/s41598-019-42687-9

**Published:** 2019-04-18

**Authors:** Amanda Brito Wardini, Lucia Helena Pinto-da-Silva, Natalia Rocha Nadaes, Michelle Tanny Nascimento, Bruno Mendes Roatt, Alexandre Barbosa Reis, Kelvinson Fernandes Viana, Rodolfo Cordeiro Giunchetti, Elvira Maria Saraiva

**Affiliations:** 1 Laboratório de Imunobiologia das Leishmanioses, Departamento de Imunologia, Instituto de Microbiologia Paulo de Góes, Universidade Federal do Rio de Janeiro, 21941-970 Rio de Janeiro, RJ Brazil; 2 Instituto de Biofísica Carlos Chagas Filho, Universidade Federal do Rio de Janeiro, 21941-902 Rio de Janeiro, RJ Brazil; 3 Instituto de Veterinária, Universidade Federal Rural do Rio de Janeiro, 23897-000 Seropédica, Brazil; 4 Laboratório de Imunopatologia, Núcleo de Pesquisas em Cíencias Biológicas, Universidade Federal de Ouro Preto, 35400-000 Ouro Preto, Minas Gerais Brazil; 5 Laboratório de Interações Célula-célula, Departamento de Morfologia, Instituto de Ciências Biológicas, Universidade Federal de Minas Gerais, 31270-901 Belo Horizonte, Minas Gerais Brazil; 6 Instituto Nacional de Ciência e Tecnologia em Doenças Tropicais (INCT-DT), Salvador, Brazil; 7 Present Address: Centro Interdisciplinar para Ciências da Vida e da Natureza, Universidade Federal da Integração Latino-Americana (UNILA), 85866-000 Foz do Iguaçu, PR Brazil

**Keywords:** Mechanisms of disease, Mechanisms of disease, Neutrophils, Neutrophils

## Abstract

Visceral leishmaniasis is a chronic disease that affects humans and dogs as well. Dogs, the domestic reservoir of *Leishmania*, play a central role in the transmission of visceral leishmaniasis, the most severe form of this disease. Neutrophils are the most abundant leukocytes in blood and interact with the parasite after infection. Here, we evaluate the effector properties of neutrophils from healthy and naturally *Leishmania infantum-*infected dogs. Our results showed that the parasite induced neutrophil extracellular trap (NET) release from neutrophils in both groups. Additionally, phagocytosis and NETs contributed differently to parasite killing by neutrophils from healthy and infected animals, and IFN-γ, IL-8, IL-4 and TNF-α production by neutrophils from both groups were differentially modulated by the parasite. Our results contribute to a better understanding of the complex role played by neutrophils in canine visceral leishmaniasis, which may favor the development of more effective therapies.

## Introduction

Canine visceral leishmaniasis (CVL), which occurs in approximately 50 countries, is a multisystemic chronic disease caused by *Leishmania infantum*^1^. Infected dogs can remain asymptomatic for many years or rapidly develop severe acute infections, developing few or multiple signs of the disease^2,3^. Dogs serve as the reservoir of *Leishmania* species and are considered the main source of infection for vectors in urban and periurban areas^4,5^. Thus, dogs play a central role in the transmission of human visceral leishmaniasis, which is the most severe form of this infection and is fatal if not treated.

Leishmaniasis begins with parasite inoculation in the host skin by phlebotomine sand flies, leading to a pool of blood in which neutrophils are the most abundant leukocytes and the first cells to be recruited to the site of infection^6^. Disease development depends on the efficiency of host mechanisms to eliminate the parasites. Neutrophils contribute to parasite destruction through an array of mechanisms, including phagocytosis, oxidative burst generation, and neutrophil extracellular traps (NETs) extrusion. NETs are fibrous webs composed of neutrophil chromatin associated with granular and cytoplasmic proteins, released into the extracellular milieu^7^. In addition to limiting parasite spread, favoring phagocytosis and killing microbes, NETs can modulate the functioning of cells present at the inoculation site^8^. *Leishmania* is a NET inducer, and some species are killed by NETs^9,10^. Notably, some *Leishmania* species have evolved mechanisms to escape NET-mediated killing such as expression of the enzyme 3′nuclease/nucleotidase, which digests the DNA scaffold^11^, protection conferred by the surface lipophosphoglycan^12^ and benefitting from the activity of the nuclease Lundep present in the vector’s saliva^13^.

It has been reported that neutrophils from *Leishmania-*infected dogs display reduced microbicidal activity and that their production of reactive oxygen species is significantly lower than that of neutrophils from healthy dogs^14^. Furthermore, the surface lipophosphoglycan of *Leishmania* promastigotes reportedly reduces neutrophil chemotactic and metabolic activities^15^, and it was recently found that neutrophils from healthy dogs in contact with *Leishmania infantum* promastigotes release NETs^16^. Although many studies have highlighted the importance of neutrophils in *Leishmania* infection in murine models^6,17–19^, the role of neutrophils in canine leishmaniasis remains underexplored. Overall, comprehensive knowledge of the parasite-neutrophil interaction, especially the role of NETs and neutrophil cytokine production, would contribute to a better understanding of the mechanisms associated with parasite immune evasion and disease establishment. Therefore, in this study we aimed to analyze the functioning of neutrophils from uninfected and naturally *L*. *infantum*-infected dogs examining NET release, killing properties and cytokine production of neutrophils.

## Methods

### Animals

Male and female mixed-breed dogs of different ages were selected on the basis of positive serological tests for CVL (ELISA and DPP^®^ Biomanguinhos/Fiocruz, Brazil), as recommended by the Brazilian Ministry of Health; samples from these dogs were kindly provided by the owners after signing an informed consent at the time of animal retrieval from the city of Governador Valadares, Minas Gerais, Brazil, an endemic area for VL. Bone marrow was aspirated from dogs with positive serology, and parasites were confirmed by NNN/LIT culture or real-time PCR. All animals were subjected to a standard quarantine protocol with broad-spectrum antihelmintic therapy and were vaccinated against rabies (Tecpar, Curitiba, PR, Brazil) before initiation of the experiments. All animals were maintained in the kennel of the Animal Science Center, Universidade Federal de Ouro Preto (UFOP), Minas Gerais, Brazil. Blood was collected from nine healthy non-infected and naturally infected dogs, which were later evaluated in a vaccination therapy protocol; their clinical characteristics are described in the results section and elsewhere^20^. Additional groups of dogs were from the Zoonosis Control Center (ZCC-BH; Belo Horizonte MG, Brazil); samples were collected immediately before euthanasia (according to Decree No. 51.838 of the Brazilian Ministry of Health). A sample of this population was evaluated, without preference with regard to breed, sex or age; in these animals, it was not possible to eliminate the interference of factors such as the presence of ecto- and endoparasites, diseases transmitted by ticks, secondary infections and nutritional deficiency. Therefore, the two groups of dogs, UFOP and ZCC-BH, were considered separately in neutrophil function analyses. The clinical criteria for selecting CVL asymptomatic and symptomatic dogs was according to Mancianti and coworkers (1988)^21^ and Reis and coworkers (2006)^22^. In addition, positive serological test for CVL, including Dual-Path Platform (DPP® CVL rapid test) and ELISA (optical density >0.200; 1:100 dilution), were defined as inclusion criteria for this study. Moreover, we associated positive serological results with parasitological tests using bone marrow aspirates to detect parasites in NNN/LIT culture or through real-time PCR. Only animals with symptoms of CVL infection, and presenting positive serological and parasitological tests for that infection were included in the study. For all animals, 20 mL peripheral blood were collected from the jugular vein into heparinized syringes and kept at room temperature until use.

### Ethics statement

The dogs used in this work were part of the study protocol approved by the Ethical Committee for the Use of Experimental Animals of the Universidade Federal de Ouro Preto, Ouro Preto, MG, Brazil, under the protocol number 2010/57. All methods were conducted in accordance with the approved guidelines of the institutional care and use committee of the Universidade Federal de Ouro Preto.

### Parasites

*L*. *infantum* (MCAN/BR/2008/OP46) promastigotes, which were isolated from an infected dog at Governador Valadares, MG, Brazil, and characterized by molecular techniques and hamster infection^23,24^. They were maintained at 26 °C in NNN-LIT medium (Sigma) supplemented with 20% heat-inactivated fetal calf serum (Criprion Biotech) and 40 μg gentamicin ml^−1^ (Schering-Plough). For all assays, promastigotes in the stationary phase of growth (5–6 days) were washed twice in PBS (pH 7.2) at 1900 *x g* for 13 min at room temperature and counted using a hemocytometer.

### Quantification of parasites in bone marrow

Bone marrow samples were collected and stored at −80 °C until processing, as previously described^25^. The parasite load was evaluated using quantitative real-time PCR (qPCR) as described^20^. Briefly, total DNA extraction was performed using Wizard SV Genomic DNA Purification System Kit (Promega, USA) according to the manufacturer’s instructions. To quantify parasite burden, primers (forward, 5′-TGT CGC TTG CAG ACC AGA TG-3′, and reverse, 5′-GCA TCG CAG GTG TGA GCA C-3′) amplifying a 90-bp fragment of a single copy of the *L*. *infantum* DNA polymerase gene (GenBank accession number AF009147) were used with the TaqMan system^26^. Standard curves were prepared for each run using known quantities of pGEM®-T plasmids (Promega, USA) containing inserts of interest. To verify the integrity of the bone marrow samples, the same procedure was carried out for the GAPDH gene (AB038240; a 115-bp fragment). Reactions were processed and analyzed using an ABI Prism 7500-Sequence Detection System (Applied Biosystems, USA). The final results are expressed as the number of amastigotes per mL of bone marrow.

### Neutrophil isolation

Peripheral blood (20 ml) collected as described above and treated with heparin as an anticoagulant (Vacutainer; BD) was layered onto a discontinuous Ficoll-Hypaque gradient (densities 1.007/1.119, Sigma) and centrifuged at 700 *x g* for 45 min at 22 °C. Neutrophils (95% purity) were recovered from the 1.119/1.007 interface, resuspended in PBS and washed by centrifugation at *400 x g* for 10 min. Cells were resuspended in RPMI 1640 without serum, and cell viability was evaluated using a 0.01% Trypan blue assay and a hemocytometer. Purity was ascertained by Giemsa staining and differential leukocyte counting.

### Immunofluorescence

Purified neutrophils (10^5^ per well) were incubated with promastigotes for 2 h at 5% CO_2_, fixed with 4% paraformaldehyde and stained with 4,6-diamidino-2-phenylindole (DAPI; 5 μg ml^−1^; Sigma) and anti-histone H2A DNA (1:800 – Cell Signaling) and anti-elastase (1:200 Calbiochem) antibodies, followed by anti-rabbit-Texas red (1: 500; Vector Laboratories) and anti-mouse-FITC (1:500; Molecular Probes) secondary antibodies. Slides were mounted using Vectashield (Vector), and images were obtained using an epifluorescence Zeiss Axioplan microscope.

### Neutrophil killing assay

Neutrophils (5 × 10^5^) were incubated with or without cytochalasin D (10 µg/ml; Sigma) and protease-free DNase-1 (100 units/ml; Fermentas Life Science); cultures were maintained for 20 min, followed by the addition of *L*. *infantum* promastigotes. After 2 h at 37 °C in 5% CO_2_, LIT medium (Sigma) supplemented with 20% heat-inactivated fetal calf serum (Criprion) was added to the preparation, and the cultures were incubated for 2 days at 26 °C. Stationary-phase promastigotes maintained as above were used as controls. Parasite growth was assessed by counting live promastigotes using a Neubauer chamber.

### Quantification of NET release

Neutrophils (10^6^/well) were incubated with or without promastigotes at a 1 neutrophil/5 parasite ratio or with 10 colony forming units (CFU) of *Escherichia coli*. After 2 h, restriction enzymes (ECOR1 and HINDIII, 20 units/ml each; BioLabs) were added to the cultures and incubated for 1 h at 37 °C. After centrifugation at 400 × *g* for 5 min, NETs in the culture supernatant were quantified using the Picogreen dsDNA kit (Invitrogen) according to the manufacturer’s instructions. Regarding *Leishmania* and *E*.*coli* stimuli, the culture supernatants were also centrifuged at 10,000 rpm for 40 seconds to remove the microorganisms. NET-DNA concentrations were calculated using herring DNA (Sigma) as a standard.

### ELISA cytokine assay

Neutrophil-*Leishmania* culture supernatants were collected after 2 h of incubation and levels of IFN-γ, IL-4, TNF-α and IL-8 were assessed by ELISA (R&D Systems) according to the manufacturer’s instructions. Briefly, plates coated with specific antibodies in PBS, pH 7.4, were blocked with PBS, 0.05% (v/v) Tween 20 and 0.1% (w/v) BSA; culture supernatants were then added to the wells. Cytokines were detected using a biotinylated monoclonal antibodies (mAb)/streptavidin-HRP with the 3,3′, 5,5′-tetramethylbenzidine (TMB) substrate, and the reaction product was evaluated at 450 nm using a plate reader (BioTek). The mean optical densities (ODs) of duplicate cultures were compared with standard curves prepared using recombinant IFN-γ, TNF-α, IL-4, and IL-8. Cytokine levels represent the differences between the ODs of test and background wells.

### Statistical analysis

Data were analyzed with GraphPad Prism 5.0 software using for two-group experiments, a non-parametric unpaired Student’s t test (one-tailed) to determine statistical significance. Comparisons of three groups and more were assessed by one-way ANOVA followed by a Tukey’s post hoc test. Results are presented as means ± SEM; p ≤ 0.05 was considered statistically significant.

## Results

Herein, we clinically classified *Leishmania infantum*-infected dogs^21,22^ according to a protocol that comprises three clinical stages, such as clinical signs, clinical pathological abnormalities and the serological status, to categorize CVL^27,28^. In fact, this detailed canine disease classification includes biomarkers that contribute to access additional clinical pathological findings to support clinicians to diagnose CVL and to provide appropriate treatment and disease monitoring. Indeed, we previously described, in investigations conducted in Brazilian endemic areas of CVL that the clinical classification proposed in this study was able to decode the clinical pathological findings according to the progression of clinical forms^29–34^. In this study, CVL was confirmed in the two groups of animals analyzed (UFOP and ZCC-BH), and the parasite load determined by qPCR varied between 100.2–24388.7 parasites/ml of bone marrow.

**Figure 1:**
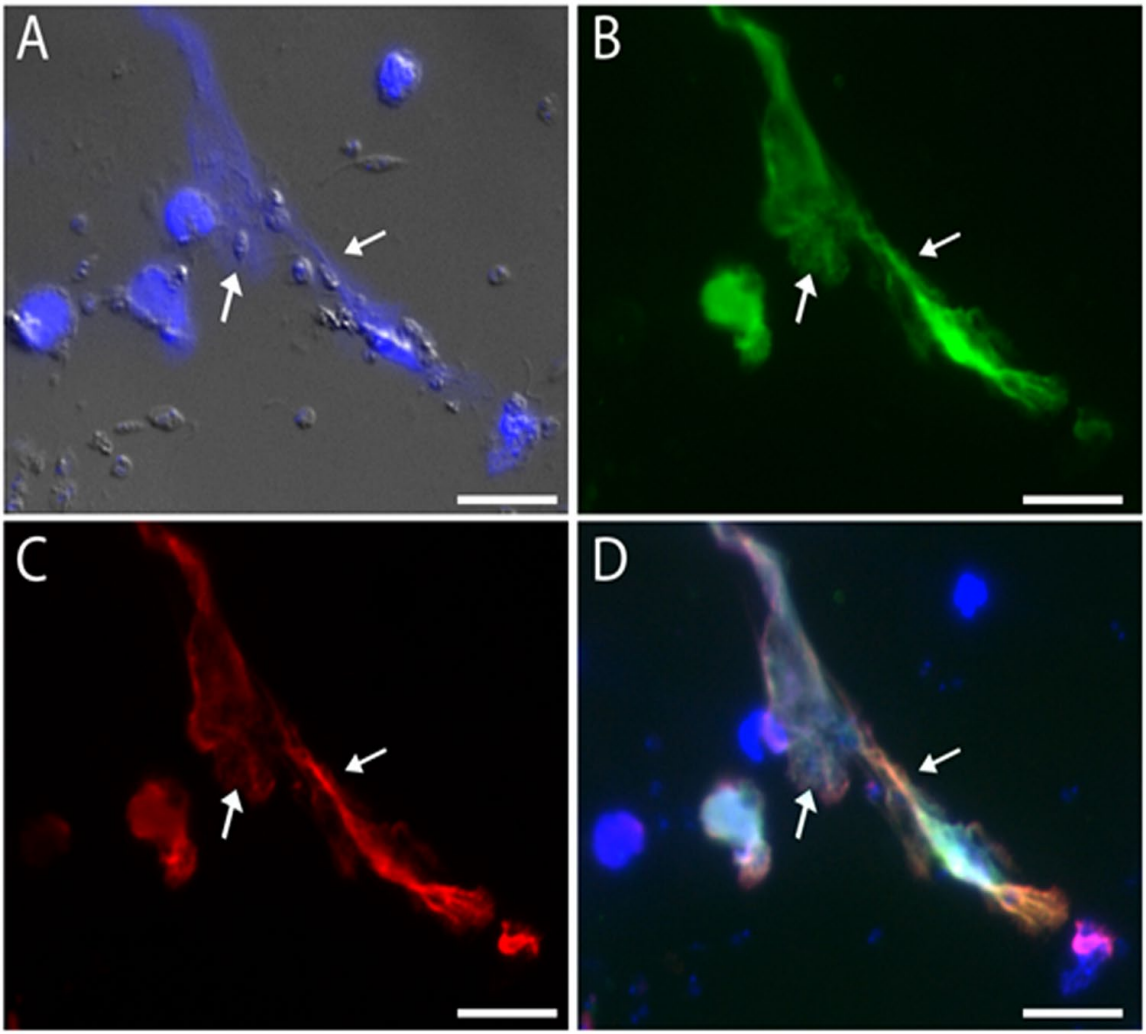
Characterization of canine NETs. Canine neutrophils were incubated with promastigotes (cell:parasite ratio of 1:5) for 2 h at 37 °C. The cells were fixed in 4% formaldehyde and stained with (**A**) DAPI or (**B**) anti-elastase or (**C**) anti-histone antibodies. (**D**) Overlay of fluorescence images. Arrows point to parasites ensnared in NETs. Bars = 20 µm.

Initially, we characterized NET extrusion by neutrophils from healthy non-infected dogs after the cells were exposed to *L*. *infantum* promastigotes. Our results showed that neutrophil incubation with promastigotes for 2 h resulted in NET release, which stained positively for DNA, histones and elastase and entrapped the parasites (Fig. 1).

**Figure 2:**
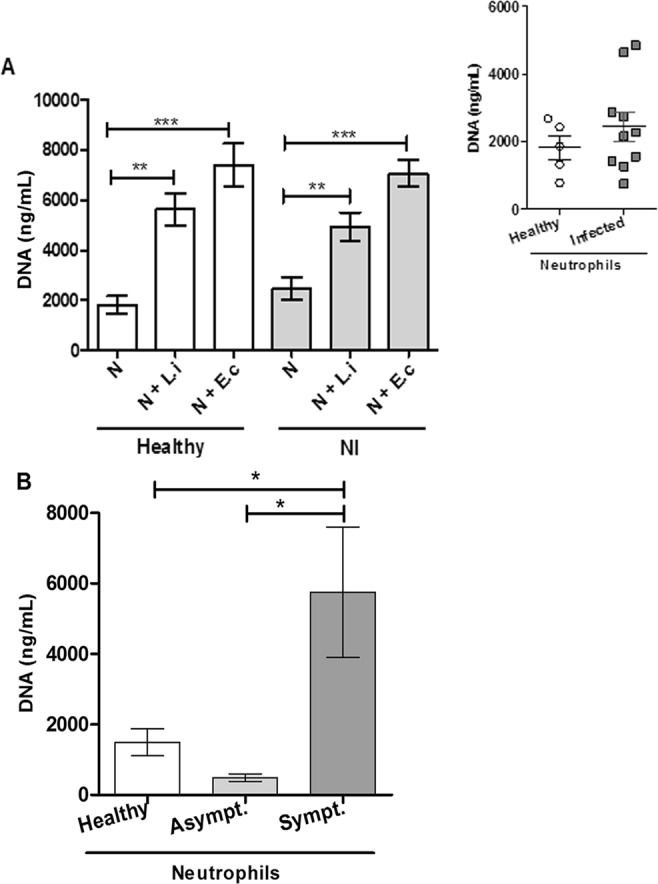
NET release *in vitro*. (**A**) Neutrophils from UFOP healthy and *Leishmania*-naturally infected dogs were stimulated with *Leishmania infantum* promastigotes (5 P:1 N) and/or *Escherichia coli* (10 CFU) for 2 h at 37 °C. (Insert) Basal NET release by neutrophils obtained from healthy and *Leishmania*-naturally infected dogs from UFOP. Insert: Basal NET release of neutrophils obtained from healthy and *Leishmania* naturally infected dogs from UFOP. (**B**) Basal NET release by neutrophils obtained from healthy dogs from UFOP and *Leishmania*-naturally infected dogs from ZCC-BH. Supernatants were recovered, and NETs were quantified using a Picogreen dsDNA kit. The results are shown as the mean ± SEM. n ≥ 5 animals/group. *p < 0.05; **p < 0.001; ***p ≤ 0.0004. NI = Naturally Infected dogs; N = neutrophils, N + L.i = neutrophils + *L*. *infantum*, N + E.c = neutrophils + *E*. *coli*, Asympt = naturally infected asymptomatic dogs; Sympt = naturally infected symptomatic dogs.

As we observed that *Leishmania* promastigotes are able to induce NET extrusion in canine neutrophils, we quantified the amounts of NETs released by neutrophils from healthy and naturally infected dogs after cell activation by a specific stimulus, such as *L*. *infantum* promastigotes, or an unrelated stimulus, such as *E*. *coli*. Our results showed increased NET release, by 3.5 and 2.6 times, by neutrophils (NØ) from healthy and naturally infected dogs stimulated with *L*. *infantum*, respectively, relative to non-stimulated control neutrophils [healthy NØ = 1.813 ± 780.9; infected NØ = 2.449 ± 1.378]. When these neutrophils were stimulated with *E*. *coli*, 2.6- and 3.6-fold increases in NET release were observed, respectively, relative to unstimulated control neutrophils (Fig. 2A). Interestingly, the amount of spontaneous NET release by neutrophils from UFOP healthy and infected dogs was not substantial (Fig. 2A, insert), whereas, basal NET release by neutrophils from ZCC-BH symptomatic infected dogs was 11.7 times higher than that from neutrophils obtained from asymptomatic infected dogs (Fig. 2B).

**Figure 3:**
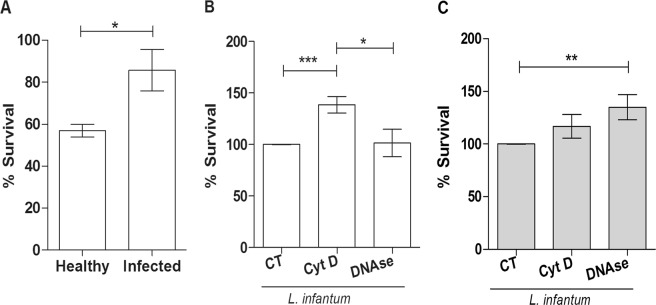
Neutrophil antimicrobial activity. (**A**) Neutrophils from healthy and naturally infected dogs were incubated with *Leishmania infantum* promastigotes (5 P:1 N) for 2 h at 37 °C. Schneider’s complete medium was then added to the cultures, and live parasites were counted after 2 days of incubation at 26 °C. The results of 9 independent experiments are shown as the mean ± SEM. *p < 0.05; ***p < 0.0001. I = Infected; H = Healthy; CT = *Leishmania* alone. (**B**) Neutrophils from healthy and (**C**) *Leishmania*-naturally infected dogs were treated with cytochalasin D and Dnase-1 and then incubated with *L*. *infantum* promastigotes for 2 h at 37 °C. Schneider’s medium was then added, and the cultures were incubated for 48 h at 26 °C. The number of viable parasites was counted using a hemocytometer. Data are expressed as the percentage of promastigote survival and shown as the mean ± SEM, *p < 0.05; **p = 0.008; ***p < 0.0001. n ≥ 9 animals/group. Cyt D = cytochalasin D. CT = untreated neutrophils.

To compare the antileishmanial activity of neutrophils from healthy and infected UFOP dogs, promastigote survival was evaluated after incubation with neutrophils. According to the results, neutrophils from healthy dogs killed 43% of the parasites compared to only 14.3% killing by neutrophils from infected dogs (healthy neutrophils killed approximately 5 times more parasites than did neutrophils from infected dogs) (Fig. 3A). Aiming to discriminate the killing mechanism, we examined the role of NETosis and phagocytosis by assessing the neutrophil-promastigote interaction using DNase to destroy NETs and cytochalasin D to inhibit phagocytosis. Incubation of *Leishmania* with neutrophils from healthy dogs demonstrated that phagocytosis inhibition increased parasite survival by approximately 40% compared to the control, though cleavage of NETs did not interfere with *Leishmania* survival compared to non-treated neutrophils (Fig. 3B). Nonetheless, for neutrophils obtained from naturally infected dogs, NET digestion increased *Leishmania* survival by 35%, though inhibition of phagocytosis did not alter parasite survival compared to the control (Fig. 3C).

**Figure 4:**
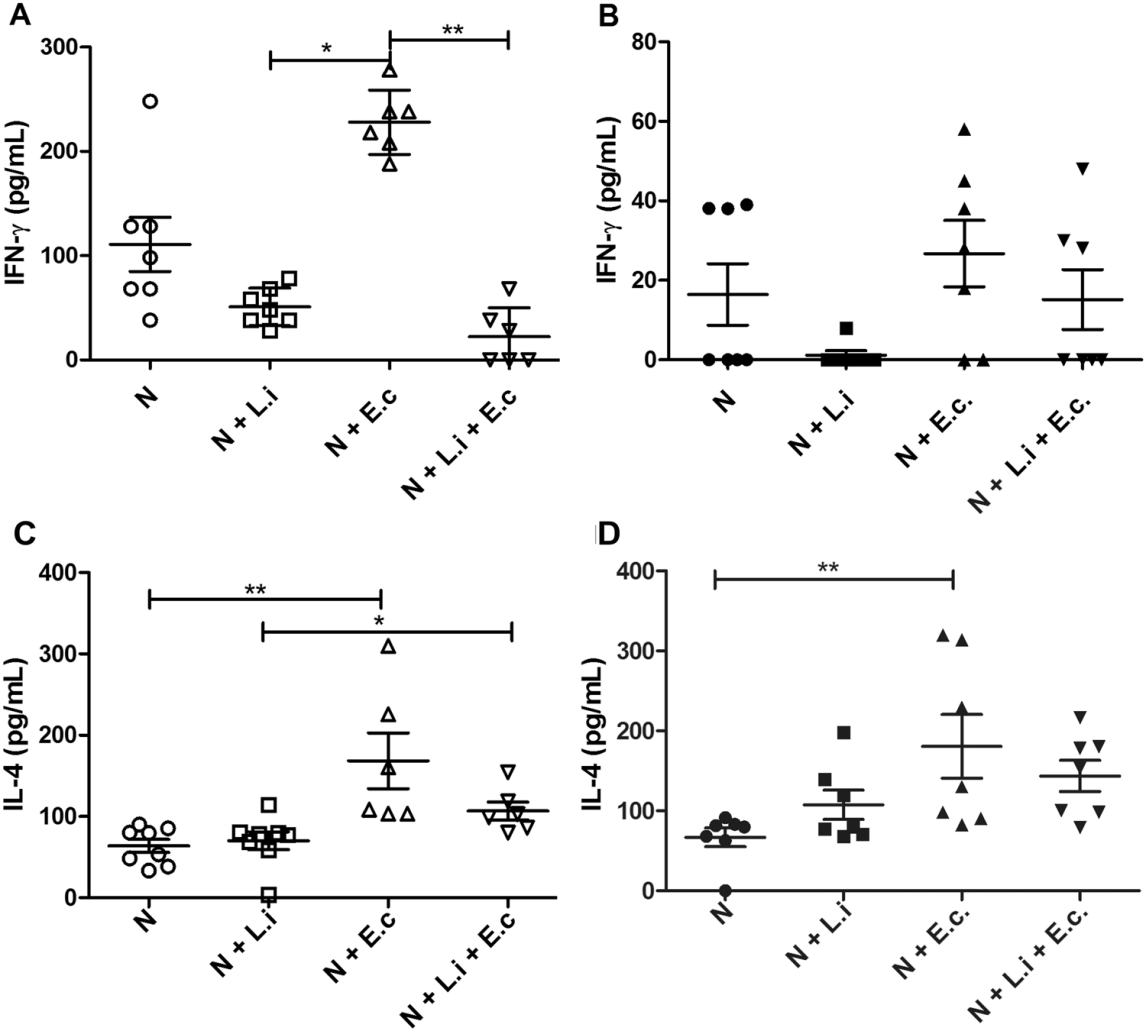
Interferon-γ (IFN-γ) and interleukin-4 (IL-4) production by neutrophils. Neutrophils obtained from healthy (**A**) or *Leishmania*-naturally infected dogs (**B**) from UFOP kennels were stimulated with *Leishmania infantum* (5 P:1 N) and/or *Escherichia coli* (10 CFU) for 2 h at 37 °C. Data are expressed as cytokine production in pg/ml and shown as the mean ± SEM. N = 7 animals/group. *p < 0.05; **p = 0.002. N = unstimulated neutrophils, N + L.i = neutrophils + *L*. *infantum*, N + E.c = neutrophils + *E*. *coli*; N + L.i + E.c = neutrophils + *L*. *infantum* + *E*. *coli*.

The cytokine profile produced by neutrophils from healthy and *Leishmania*-infected dogs stimulated with *L*. *infantum* and/or *E*. *coli* was also evaluated, revealing that *L*. *infantum* interaction with healthy neutrophils decreased IFN-γ production by 2.3-fold compared to non-stimulated neutrophils, but not significantly. However, after *E*. *coli* stimulation, these same neutrophils significantly responded with double the production (2.3-fold over control) of this cytokine. Interestingly, addition of *Leishmania* to the *E*. *coli*-neutrophil culture significantly inhibited IFN-γ production (Fig. 4A), and neutrophils from *Leishmania*-infected dogs produced 6.75-fold lower levels of IFN-γ than did neutrophils from healthy dogs (Fig. 4A,B). Although not statistically significant (likely because of the different IFN-γ production among unstimulated healthy dogs), *L*. *infantum* exposure completely abolished IFN-γ production compared to non-stimulated neutrophils, and no increase in IFN-γ production was observed after stimulation with *E*. *coli* alone or with *Leishmania* (Fig. 4B).

Regarding IL-4, neutrophils from both healthy and infected dogs produced similar basal levels of IL-4, which increased only with the *E*. *coli* stimulus (Fig. 4C,D). Interestingly, *L*. *infantum* was unable to stimulate IL-4 production in both groups of neutrophils.

**Figure 5:**
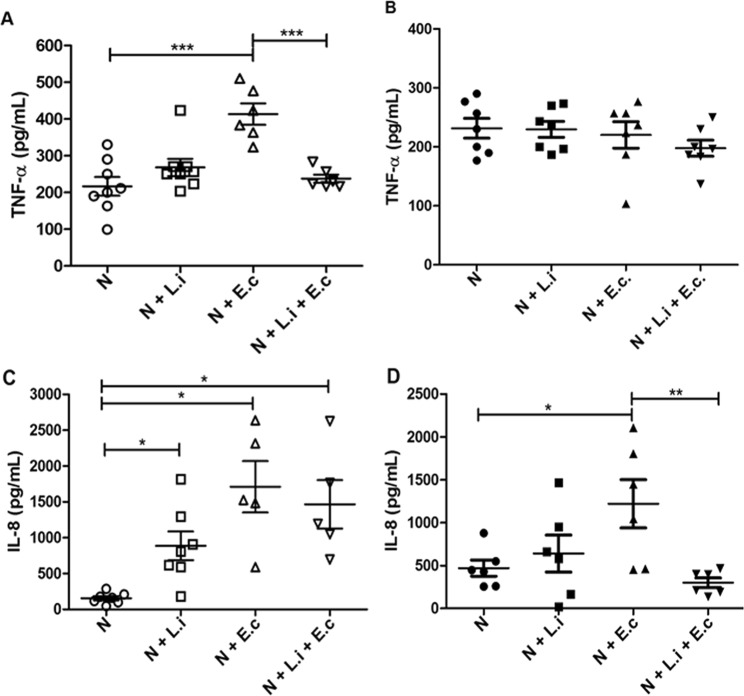
Tumor necrosis factor-α (TNF-α) and interleukin-8 (IL-8) production by neutrophils. Neutrophils obtained from healthy (**A**,**C**) or *Leishmania*-naturally infected dogs (**B**,**D**) from UFOP kennels were stimulated or not with *Leishmania infantum* (5 P:1 N) and/or *Escherichia coli* (10 CFU) for 2 hours at 37 °C. Data are expressed as cytokine production in pg/ml and shown as the mean ± SEM. n = 7 animals/group. *p < 0.05; **p < 0.005; ***p ≤ 0.0003. N = unstimulated neutrophils, N + L.i. = neutrophils + *L*. *infantum*, N + E.c = neutrophils + *E*. *coli*; N + L.i + E.c = neutrophils + *L*. *infantum* + *E*. *coli*.

TNF-α production by neutrophils from healthy dogs was 1.7-fold higher after stimulation with *E*. *coli* compared to non-stimulated neutrophils, and co-culture with *L*. *infantum* reduced this production by 44% (Fig. 5A). No significant difference was observed in TNF-α production between unstimulated or *Leishmania-*stimulated neutrophils (Fig. 5A), and neutrophils obtained from infected dogs produced similar levels of TNF-α compared with neutrophils from healthy animals (Fig. 5A,B). Moreover, TNF-α production by *Leishmania*-infected dogs was not modulated by any of the stimuli tested (Fig. 5B).

Neutrophils from healthy dogs produced IL-8 in response to *L*. *infantum*, *E*. *coli* or both stimuli together (Fig. 5C), whereas neutrophils from *Leishmania*-infected dogs responded only to the *E*. *coli* stimulus (Fig. 5D). Interestingly, basal levels of IL-8 production were 2.8 times higher in neutrophils from infected dogs compared to those from healthy animals (Fig. 5C,D). In addition, co-stimulation of neutrophils from infected dogs with *L*. *infantum* and *E*. *coli* reduced IL-8 production 4-fold in relation to stimulation with *E*. *coli* alone.

## Discussion

Early interaction of neutrophils with *Leishmania* is one of the events of this infection that is well established in the literature, and this step has also been demonstrated in experimental canine infection^16,35^. Infiltrating neutrophils kill *Leishmania* parasites through phagocytosis, the oxidative burst and immune response modulation via cytokine production^14,16,35,36^. More recently, an additional mechanism has been added with the description of NETosis induced by *Leishmania* in human and mouse neutrophils^9,12,18^. Although it has been reported that neutrophils from dogs release NETs upon exposure to different stimuli^37,38^, studies comparing NET formation among infected and non-infected dogs are still lacking.

Here, we show that resting neutrophils stimulated by *L*. *infantum* promastigotes release NETs composed of a DNA scaffold decorated with elastase and histones, which is different from previous findings^16^. This difference may be attributed to the dog breed, sex, age and/or method(s) used for analysis. NET quantification demonstrated that neutrophils from both healthy and naturally infected dogs released similar amounts of NETs upon stimulation with promastigotes and with *E*. *coli*. Our results showed at least 3-fold increased NET release by neutrophils from healthy dogs upon contact with *L*. *infantum* promastigotes. In addition, spontaneous NET release was similar between the two UFOP groups of animals. Interestingly, a significant difference in spontaneous NET extrusion was observed by neutrophils from VL symptomatic dogs from the ZCC-BH. Regardless, these differences cannot be attributed to VL as the only cause because canine visceral leishmaniasis is a chronic infection and may occur in association with other diseases that were not investigated in the ZCC-BH animals. Nevertheless, the ZCC-BH asymptomatic infected dogs presented much lower spontaneous release of NETs.

Leishmanicidal activity has been demonstrated for NETs generated by human neutrophils^9^. In our work with canine neutrophils, we observed higher parasite survival after incubation with neutrophils from naturally infected dogs compared to neutrophils from healthy dogs, indicating a lesser ability of the former cells to control *Leishmania in vitro*. By analyzing the mechanisms responsible for this difference, we showed that for healthy animals, phagocytosis appears to be more important for *Leishmania* control *in vitro* than does NET release because only treatment with cytochalasin D enhanced survival of the parasite. A similar observation was reported for human neutrophils, whereby DNase treatment did not increase *Leishmania donovani* survival *in vitro*^12^. Regardless, we found that NETs were crucial for neutrophils from naturally infected dogs to control *Leishmania* because the addition of DNase to the culture significantly promoted parasite survival. It has been reported that neutrophils from naturally *Leishmania*-infected dogs display reduced killing ability according to the disease stage^14,34,36^. Moreover, a study involving a human visceral leishmaniasis patient found a decreased ability of neutrophils to release NETs, evidencing altered neutrophil function for visceral leishmaniasis patients^39^.

As cytokines are key mediators for determining the type of immune response invoked, it is important to characterize the cytokines produced by neutrophils, which are among the first cells to interact with *Leishmania* parasites. Moreover, some studies have already shown that NETosis can be induced by cytokines^7^ and that cytokines are released during NETosis^40^; thus, we evaluated cytokines released upon neutrophil-parasite interaction. We found that neutrophils from unstimulated naturally infected animals produced a smaller amount of IFN-γ in relation to healthy animals and that *L*. *infantum* decreased IFN-γ levels in neutrophils from both healthy and infected dogs. Interestingly, this inhibitory effect of promastigotes was observed even when IFN-γ production was stimulated by *E*. *coli* in neutrophils from healthy animals. It has been reported that for asymptomatic and symptomatic naturally infected dogs, neutrophils stimulated with a soluble *Leishmania* antigen produced similar high levels of IFN-γ^34^. Although neutrophils were not analyze, low levels of IFN-γ mRNA were reported for mononuclear cells from symptomatic naturally infected dogs^41,42^, and in another study, higher levels of IFN-γ mRNA were detected in mononuclear cells from similarly infected dogs after stimulation with a mitogen or soluble *Leishmania* antigen^43^.

The association between TNF-α and IFN-γ is responsible for the activation of macrophages and death of amastigotes in asymptomatic dogs^44^. Compared with asymptomatic dogs, mononuclear cells from symptomatic dogs produce lower levels of IFN-γ and TNF-α mRNA after stimulation with the *Leishmania* antigen^45^. In our study, a difference in TNF-α basal production was observed between neutrophils from naturally infected and healthy dogs, and for the group of healthy animals, parasites downregulated the TNF-α release caused by *E*. *coli*. Because IFN-γ and TNF-α are cytokines that participate in macrophage activation for nitric oxide (NO) production, inhibition of these cytokines leads us to infer that the parasite is able to modulate neutrophil cytokines, even when levels of these cytokines are stimulated, for example, by *E*. *coli*.

Although it has been shown that neutrophils produce IL-8 and that this cytokine stimulates NET release^7^, the role of IL-8 in neutrophils from dogs with leishmaniasis has not yet been evaluated. Our results demonstrate that promastigotes stimulated IL-8 production by neutrophils from healthy dogs. Although the parasites did not induce significant IL-8 release from neutrophils of naturally infected dogs, these neutrophils released 3.2 times more basal IL-8 than did neutrophils from healthy animals. This elevated IL-8 response by cells from infected dogs may participate in NET release because IL-8 has also been described as a NET stimulus^7^. Indeed, IL-8 production was significantly increased in neutrophils from healthy dogs stimulated by parasites but not after parasite stimulation of neutrophils from infected dogs. Interestingly, parasite stimulus significantly decreased the IL-8 release induced by *E*. *coli* in neutrophils from naturally infected animals, an effect that was not observed for neutrophils from healthy dogs. Further studies are necessary to understand the role of IL-8 in *Leishmania*-induced NET release.

Overall, the role of IL-4 in canine visceral leishmaniasis remains controversial. Increased expression of IL-4 mRNA in mononuclear cells after mitogen stimulation in asymptomatic and symptomatic animals has been reported^46^. However, others have described that no IL-4 mRNA expression was observed in mononuclear leukocytes from dogs with different clinical stages of the disease^47^, and reduced IL-4 production has been found for neutrophils from asymptomatic seronegative compared to asymptomatic seropositive and symptomatic animals^34^. Increased levels of IL-4 production by neutrophils stimulated with soluble *Leishmania* antigens can occur in human visceral leishmaniasis patients^48^. In fact, higher levels of IL-4 are considered a hallmark of *L*. *infantum*-naturally infection in dogs^49^, and reduction in IL-4 levels after vaccine immunization against CVL has been considered a biomarker for protection against *Leishmania* infection^50,51^. In contrast, it has been shown that after *Leishmania* stimulation, neutrophils from asymptomatic dogs with positive serological and PCR tests and symptomatic dogs exhibited a high frequency of IFN-γ production^34^. In this study, we did not observe any significant difference in the production of IL-4 among neutrophils from healthy and naturally infected dogs, with both groups of neutrophils responding similarly to the parasite stimulus and exhibiting the same basal level of cytokine release. Interestingly, *E*. *coli* resulted in enhanced IL-4 production by both groups of cells.

The IFN-γ/IL-4 ratio for neutrophils from healthy dogs was 1.7, and when these cells were stimulated by the parasite, the ratio decreased to 0.7. However, the IFN-γ/IL-4 ratio for neutrophils from naturally infected dogs was 0.3, which fell to 0.009 when cells were stimulated by *Leishmania*, suggesting that the parasite may subvert the cytokines produced by neutrophils to favor its survival in the vertebrate host.

In conclusion, our findings unveil the effector properties of neutrophils from healthy dogs and those naturally infected with *Leishmania*, evidencing NET release, killing mechanisms and cytokine production by these cells. A better understanding of the complex role played by neutrophils in visceral leishmaniasis may contribute to more efficient therapies.

## Data Availability

The authors declare that all data are available.
